# Preparation of Carbon Nanotube/TiO_2_ Mesoporous Hybrid Photoanode with Iron Pyrite (FeS_2_) Thin Films Counter Electrodes for Dye-Sensitized Solar Cell

**DOI:** 10.1038/srep27052

**Published:** 2016-05-31

**Authors:** Bayram Kilic, Sunay Turkdogan, Aykut Astam, Oguz Can Ozer, Mansur Asgin, Hulya Cebeci, Deniz Urk, Selin Pravadili Mucur

**Affiliations:** 1 Department of Energy Systems Engineering, Faculty of Engineering, Yalova University, 77100, Yalova, Turkey; 2 Department of Physics, Faculty of Science and Art, Erzincan University, Erzincan, 24100 Turkey; 3 Department of Aeronautical Engineering, Istanbul Technical University, Maslak 34469, Istanbul, Turkey; 4 Department of Chemical Metallurgical Engineering, Istanbul Technical University, Maslak 34469, Istanbul, Turkey; 5 TUBITAK MAM Institute of Materials, Gebze, Kocaeli, 41400 Turkey

## Abstract

Multi-walled carbon nanotube (MWCNT)/TiO_2_ mesoporous networks can be employed as a new alternative photoanode in dye-sensitized solar cells (DSSCs). By using the MWCNT/TiO_2_ mesoporous as photoanodes in DSSC, we demonstrate that the MWCNT/TiO_2_ mesoporous photoanode is promising alternative to standard FTO/TiO_2_ mesoporous based DSSC due to larger specific surface area and high electrochemical activity. We also show that iron pyrite (FeS_2_) thin films can be used as an efficient counter electrode (CE), an alternative to the conventional high cost Pt based CE. We are able to synthesis FeS_2_ nanostructures utilizing a very cheap and easy hydrothermal growth route. MWCNT/TiO_2_ mesoporous based DSSCs with FeS_2_ CE achieved a high solar conversion efficiency of 7.27% under 100 mW cm^−2^ (AM 1.5G 1-Sun) simulated solar irradiance which is considerably (slightly) higher than that of A-CNT/TiO_2_ mesoporous based DSSCs with Pt CE. Outstanding performance of the FeS_2_ CE makes it a very promising choice among the various CE materials used in the conventional DSSC and it is expected to be used more often to achieve higher photon-to-electron conversion efficiencies.

Dye-sensitized solar cells (DSSCs) have been intensively studied with a growing demand as potential alternatives for the next generation solar cells due to their low cost and eco-friendly production, easy processing, and relatively high energy conversion efficiency when compared with conventional solar cells^1^,^2^. In contrast to the conventional solar cells, which relies on high purity substrates grown at very high temperatures using high cost processes in a specially designed environments such as clean room, DSSCs do not require such costly and complex processes and can be prepared in a simple laboratory environment without much concern on materials purity and ambient atmosphere^3^. A typical DSSC consists of TiO_2_ mesoporous structures as photoanode deposited on fluorine doped tin oxide (FTO) coated glass substrate and sensitized by dye molecules and Pt coated FTO counter electrode (CE) with a I^−^/I_3_^−^ redox electrolyte filled in between photoanode and CE^4^. Under solar irradiation, excited electrons in the Ruthenium-dyes are injected into the conduction band of TiO_2_ nano-particles and diffuse into the FTO/TiO_2_ interface and eventually are extracted to an external load^5^. The extracted electrons flow through the load and reach the CE. I^−^/I_3_^−^ redox electrolyte accepts the electrons from Pt CE and those are transferred to the dye molecules to refill the holes in the HOMO^6^. Various methods are being investigated to increase the solar conversion efficiency of DSSCs^7^. Although the efficiency of the DSSCs has reached up to 11–12%, efforts to improve the photoelectron collection efficiency have been only reported in a few researches^8^,^9^. The main problems in DSSCs are described as inefficient charge separation and electron transport, carrier recombination at surface states, cell instability, and inefficient adsorption of dye molecules on the FTO/TiO_2_ surface^10^,^11^. In recent years, one dimensional (1D) nano-semiconductors with different morphologies such as nanorods, nanowires, nanotubes, nanobelts and nanosheets have been extensively used to improve the electron transport characteristic and to reduce the charge recombination in DSSCs^12^,^13^, as well. Nanostructure based photoanodes grown by various type of materials in different nanostructure morphologies is another approach to increase the electron collection probability. Carbon based nanomaterials such as carbon nanotube and graphene can improve the overall performance of the cell through better electron transport properties caused by well-defined band alignment between adjacent layers^14^,^15^. Carbon nanotubes (CNTs) with high electrical conductivity and very large surface area are an ideal substance for improving the charge transport and therefore the photo-generated current in photoanode of DSSCs^16^,^17^. CNTs have been widely used in different layers of DSSCs such as in photoanode with the mixture of TiO_2_, in counter electrode and solid state electrode for different applications^18^. However, it has been shown that the incorporation of CNTs in photoanode is the most effective way to improve the cell efficiencies due to better charge transport properties^19^,^20^. MWCNT/TiO_2_ photoanode based DSSC exhibits not only the advantage of high quality interface between CNTs and titania matrix, but also leads to the coexistence of dual pores which provide high surface area which is necessary for the dye adsorption^10^,^11^,^12^,^13^,^14^,^15^,^16^,^17^,^18^,^19^,^20^,^21^. Besides photoanode components, CE also plays an important role to increase the solar conversion efficiency of DSSCs. In conventional DSSCs, Pt-coated FTO (Pt:FTO) and carbon are usually used as CE due to their exceptional catalytic activity, and high electrical conductivity^22^. Although DSSCs designed with Pt CE shows a good performance, Pt sources are scarce and increasing the cost of DSSCs^23^. Carbon based CE, on the other hand, is cheaper than Pt, but its shows much lower performance when used as CE and therefore this drives the scientific community to look for other high performance and low cost materials to be utilized as CE in DSSCs^24^. Recent works shows that FeS_2_ nanostructures exhibit a high surface area which is beneficial for the catalytic reaction and the solar power conversion efficiency. FeS_2_ with a optical energy band gap of 0.95–1.5 eV has attracted much interest with its inherent advantages such as excellent photo absorption, environmental compatibility, high electron mobility, large optical absorption coefficient, non-toxic and high quantum efficiency (>80%) in both fundamental research and practical applications^25^,^26^. Since FeS_2_ is thermodynamically stabile in the corrosive I^−^/I^−^_3_ redox liquid electrolyte this material has been extensively studied as possible alternative for Pt based CE in DSSCs^27^.

In this paper, MWCNT/TiO_2_ mesoporous photoanode is studied to investigate the effect of CNT based TiO_2_ mesoporous on the DSSC performance and the enhancement capability of A-CNT/TiO_2_ photoanode on solar conversion efficiency in DSSC is compared with a conventional TiO_2_ photoanode. The Influence of MWCNT/TiO_2_ photoanode on solar conversion efficiency of DSSC is compared with a conventional TiO_2_ photoanode and result shows that efficiency is increased from 6.51% to 7.00% due to the enhanced light absorption and electron transport across the TiO_2_ mesoporous based thin films. Besides photoanode components, we also demonstrate that FeS_2_ nanostructures are very promising alternative (η = 7.27%) to the Pt CE and it can be used as an efficient and low cost CE in DSSCs. Structural and optical characterizations are carried out using X-ray diffraction spectroscopy (XRD), scanning electron microscopy (SEM), energy dispersive spectroscopy (EDS), Raman spectroscopy and UV-Vis analyses.

## Experimental

### Preparation of A-CNT/TiO_2_ Hybrid Photoanodes

Multiwalled CNTs (MWCNTs) were grown by thermal catalytic chemical vapor deposition (CVD) on silicon wafers using a thin catalyst layer of Fe/Al_2_O_3_ (1/10 nm) deposited by electron beam evaporation. MWCNT growth was performed in a quartz tube furnace (22 mm ID) at atmospheric pressure using ethylene as the carbon source. The nominal growth temperature was 750 °C, yielding an average growth rate (including nucleation and growth) of ~2 *μ*ms^−1^. Typically, CNT arrays are grown on 1 cm^2^ Si wafers, resulting in well-aligned CNTs (MWCNTs) with densities of 10^9^–10^10^ CNTs cm^−2^. As-grown CNT arrays have 1% volume fraction. The CNT arrays were kept face down in a vacuum chamber, facing the oxidizing agent (Details are described in our early work)^28^. As-grown vertically-aligned multi-walled carbon nanotubes (MWCNTs) were dispersed onto a FTO (F:SnO_2_) substrate (with a Sheet resistance of 14 Ω/sq) via contact printing. Aqueous solution of 1 mM titania (IV) oxide, 10 ml HCl and 40 ml deionized water were prepared and placed into a Teflon-lined stainless steel autoclave. 8 ml ammonia (28%) was added into the solution to adjust pH >4.5. MWCNT/FTO substrates were immersed into the solution and then heated to 175 °C for 24 hours. After hydrothermal growth processes, the whole system was allowed to cool down to the room temperature (RT) and substrate was blow dried by N_2_. Finally, the TiO_2_ mesoporous film was obtained on the MWCNT/FTO substrate and the sample were placed into a furnace at 450 °C for 40 min to minimize the defects.

### Preparation of Iron Pyrite (FeS_2_) Counter Electrodes

FeCl_3_.6H_2_O (Sigma-Aldrich) and Sulfur powder (%99.98) were used as precursor. 0.3 g iron chloride and 0.1 g sulfur were prepared in 40 ml DI water. The pH of the mixture was adjusted to 11 by adding a specific amount of Ammonia (%28). The solution was kept under magnetic stirring at RT for 1 h. The mixture was placed and sealed in a Teflon-lined stainless steel autoclave after immersing the FTO substrate into the solution. After that the autoclave was placed into an oven at 175 °C and kept there for 8 h to synthesis FeS_2_ materials. A thin layer of FeS_2_ nanostructures was obtained on the FTO substrate and washed with distilled water and ethanol several times to remove the excess of polymer and ion contaminants, and dried at 75 °C for 6 h in a vacuum oven. Finally, the film was sulfurized at 400 °C for 1 hr in nitrogen atmosphere.

### Device Fabrication of DSSCs based on A-CNT/TiO_2_ Hybrid Photoanodes and FeS_2_ Counter Electrode

Hybrid DSSCs based on MWCNT/TiO_2_ were prepared by adsorption of cis bis (isothiocyanato)bis(2,20-bipyridyl-4,40-dicarboxylato)-ruthenium(II)bis tetrabutylammonium (N719) dye onto the surfaces of the prepared photoanode for 6 h. It is important to note that the substrates were heated to 100 °C for 30 min before immersing into a 0.5 mM solution of the N719 dye. After 6 h the samples were taken out, rinsed with acetonitrile, and dried with nitrogen gas. The dye-sensitized MWCNT/TiO_2_ photoanode and FeS_2_ counter electrode were sandwiched together using a 20 μm thick transparent Surlyn film (Meltonix 1170, Solaronix). The electrolyte, which consists of 0.5 M tetrabutylammonium iodide, 0.05 M I_2_ and 0.5 M 4-tertbutylpyridine in acetonitrile, was injected between two electrodes and well distributed via capillary action. The active electrode area was typically 0.25 cm^2^ for all type of cells studied in this work.

### Characterization of the device

The structural and chemical characterizations of the nanostructures on each prepared sample were analyzed using Philips XL30 ESEM-FEG scanning electron microscope equipped with an EDAX energy dispersive X-ray spectroscopy detector. Crystal structure analysis was carried out using X-ray diffraction (XRD; Rigaku D/Max-IIIC diffractometer) with 1.54 Å Cu-Kα radiation and 2θ range of 20–80°. Absorption measurements were performed using a Perkin-Elmer UV-VIS Lambda 2S spectrometer. The Raman scattering measurements were performed using a micro Raman Renishhaw 2000 system with an excitation source of 514.5 nm at RT. The infrared spectra were recorded using Fourier-transform infrared (FTIR) spectrometer, Perkin Elmer, in transmittance mode at 450–4000 cm^−1^. Photocurrent density versus voltage (J-V) data were recorded using a Keithley 175A digital multimeter using a 0.01 V/s voltage ramp rate and an AM 1.5 solar simulator. The light source was a 250 W tungsten halogen lamp calibrated to irradiate the samples at 100 mW/cm^2^ using a radiometer (IL1700, International). The incident photon current efficiency (IPCE) was measured with a spectral resolution of 5 nm using a 300W xenon lamp (Newport/Oriel). A reference scan of incident photon flux was taken using a calibrated Si photodiode.

## Results and Discussion

### The Effect of A-CNT Photoanodes on DSSC Performance

**Figure 1:**
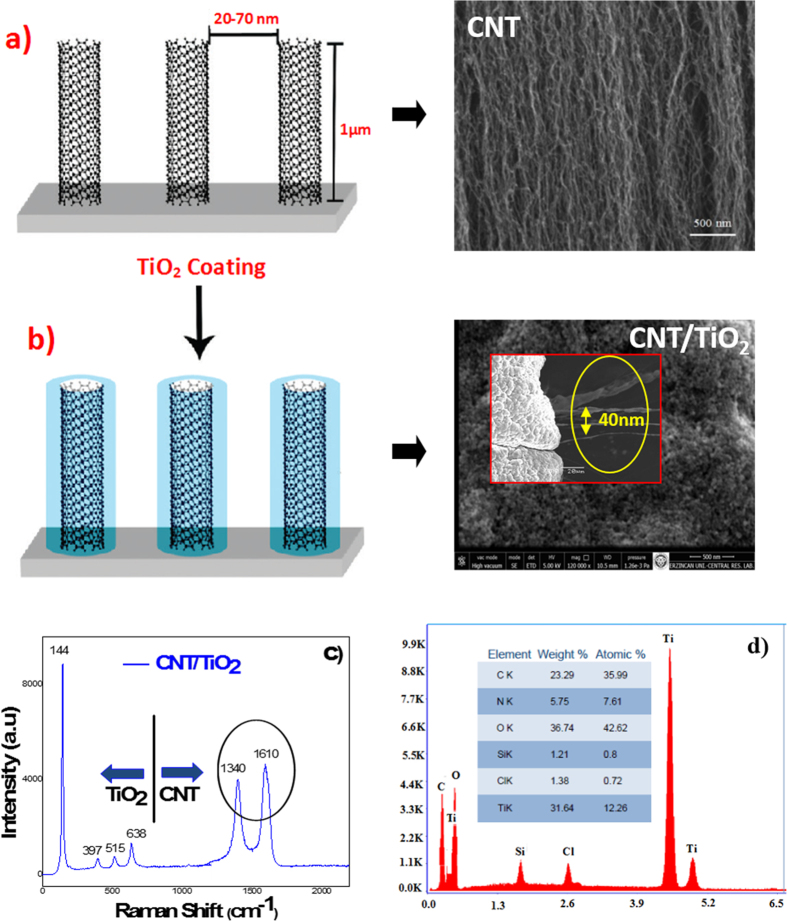
Top view and cross-sectional SEM images with corresponding schematic illustration of (**a**) CNT and (**b**) CNT/TiO_2_ hybrid (network) structures (**c**) Raman and (**d**) EDAX spectra of CNT/TiO_2_ complexes after annealing at 400 °C.

Top view and cross sectional SEM images of A-CNT/TiO_2_ mesoporous photoanode are shown in Fig. 1(a,b) at different magnifications. ~1 μm long CNT arrays were coated with TiO_2_ mesoporous structures and this is the key starting point of forming higher-phase hybrid system. Figure 1(b) shows that the FTO/MWCNT/TiO_2_ hybrid system with inter-CNT spacing of 20–70 nm and it is clearly seen that CNT arrays were uniformly coated with TiO_2_ without disturbing the CNTs’ morphology. It is demonstrated that extremely high aspect ratio CNT arrays can be coated with TiO_2_ mesoporous structures having about 30–50 nm pore radius. As seen in the SEM images, in the case of the TiO_2_ coating the distance between the TiO_2_ coatings 1% volume fraction CNTs have an average inter-CNT spacing of 70 nm, whereas TiO_2_-coated CNTs have 40 nm of intertube spacing because of the 30 nm coating on each CNT. As seen in SEM images, the A-CNTs are well-dispersed within TiO_2_ mesoporous thin film, and a good contact between nanotubes and TiO_2_ mesoporous is achieved. This incorporation is very important for introducing an alternative electrical conduction pathway into the FTO/MWCNT/TiO_2_ hybrid system. With high electrical conductivity of MWCNTs, it’s expected to observe enhanced electron transport rate and life time in the TiO_2_ mesoporous structure which eventually increase the solar conversion efficiency. The Raman spectroscopy measurements are carried out to further characterize the MWCNT/TiO_2_ nanostructure complex. Raman scattering spectrum clearly identifies the phase of the TiO_2_ mesoporous on the basis of its Raman band of Eg mode at 144 cm^−1^. It is known that the Eg mode corresponds to O-Ti-O bending type vibration. Three peaks at 397, 515, and 638 cm^−1^ in the Raman spectra of CNT/TiO_2_ in Fig. 1(c) can be associated with the B1g (1), A1g+B1g (2), and Eg (2) modes of TiO_2_, respectively. The other two peaks around 1340 and 1610 cm^−1^ are associated with the characteristic D-band and G-band of CNTs corresponding to the disordered mode and tangential mode, respectively. Chemical characterization of the samples is carried out by Energy-dispersive X-ray spectroscopy (EDX). The EDX spectra of MWCNT/TiO_2_ hybrid system is shown in Fig. 1(d) and shows the presence of major elements of C and O with strong Ti peaks. EDX quantitate analysis result of the MWCNT/TiO_2_ hybrid film is presented in the inset of Fig. 1(d). In general, most of the MWCNT/TiO_2_ hybrid film samples contain oxygen and titanium as major elements with small quantities of carbon.

**Figure 2:**
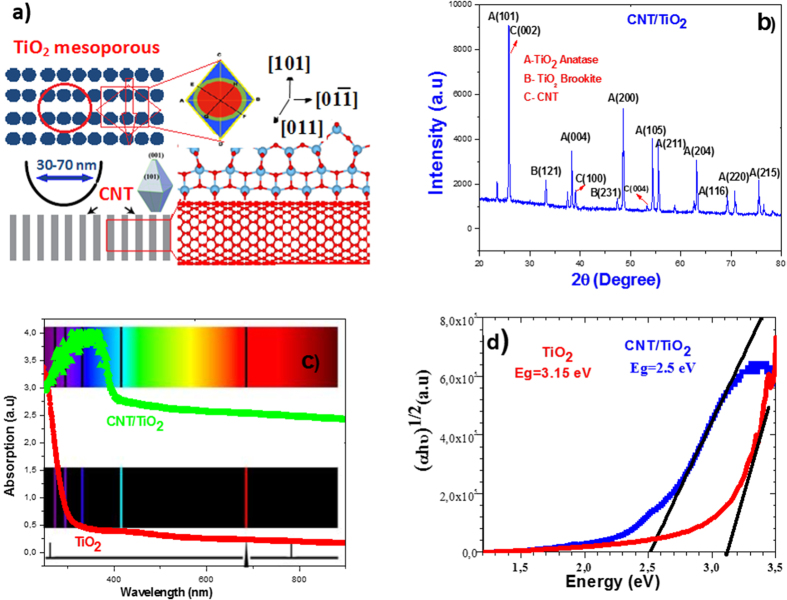
(**a**) Crystal structure and (**b**) XRD pattern of CNT/TiO_2_ hybrid structures (**c**) Absorbance spectra and (**d**) Tauc plot of CNT/TiO_2_ hybrid structures to determine the optical band gaps at room temperature.

**Figure 3:**
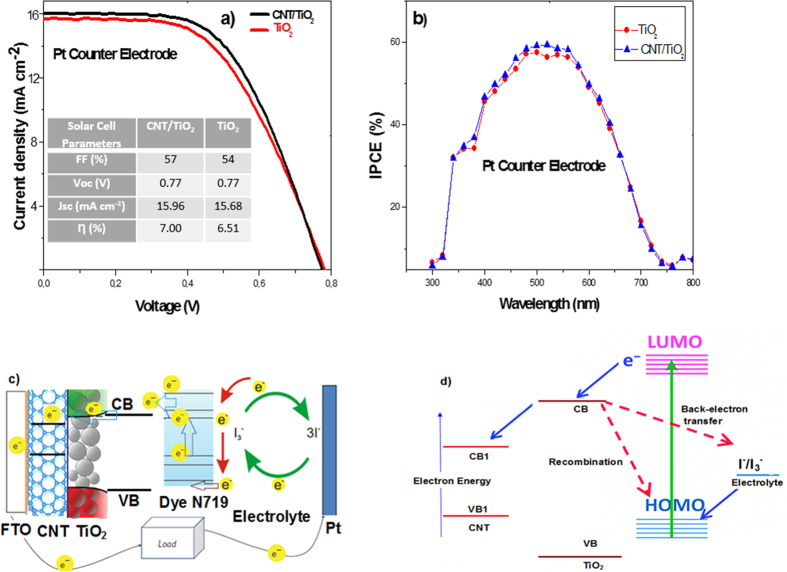
(**a**) Current density-voltage (J-V) characteristics of MWCNT/TiO_2_ and TiO_2_ photoanode with Pt CE based dye-sensitized solar cells under 1-Sun AM 1.5G solar irradiance (CNT wt% is 1 and the film thickness is about 15 μm) (**b**) IPCE spectra of the corresponding cells, (**c**) Schematic view of electron (e^−^) diffuse transport behavior in CNT/TiO_2_-network hybrid electrode, (**d**) Energy level diagrams of the cell with possible loss mechanisms.

XRD characterization is used to determine the crystallographic structure of the MWCNT/TiO_2_ hybrid system. In Fig. 2(a,b), some well-defined diffraction peaks at 25.8°, 33.1°, 39.0°, 48.5°, 54.3°, 55.4°, 63.0°, 69.4°, 70.6°, and 75.4° can be observed. These peaks are assigned to the (1 0 1), (121), (231), (2 0 0), (1 0 5), (2 1 1), (2 0 4), (1 1 6), (2 2 0), and (2 1 5) reflections of crystalline anatase and brookite phase according to No. 21-1272 JCPDS, respectively. The result indicated that the main component of the thin film is anatase TiO_2_. Among all diffraction peaks of MWCNT/TiO_2_, (002) peak for CNT and (101) peak for TiO_2_ mesoporous structures are thermodynamically the most stable due to the lowest surface energy. The peaks of 25.8°, 38.3°, 52.9° are associated with the typical peaks of CNT in (002), (100) and (004) directions, respectively. XRD characterization also shows brookite structures for TiO_2_ mesoporous with CNT incorporation. Since carbon is very strong reducing agent, it would be likely to enhance the small transformation of anatase to brookite structures. The UV-Vis absorption spectra of the pure TiO_2_ and MWCNT/TiO_2_ nanostructures are shown in Fig. 2(c,d). As seen the MWCNT/TiO_2_ exhibits a stronger visible light absorption than the pure TiO_2_ mesoporous structures. The absorption edge of these also shifted towards the longer wavelength side, which indicates an ability of the hybrid systems to be photoactivated under the visible light irradiation. Furthermore, this may be referred to the mesoporous surface of the MWCNT/TiO_2_ hybrid system and it is an advantage to absorb broader range of the solar spectrum. Figure 3(d) shows the (αhν)^1/2^ versus photon energy which was used to calculate the bandgap energy of MWCNT/TiO_2_ and pure TiO_2_ structures. The result indicates that the bandgap (Eg) of TiO_2_ mesoporous is 3.15 eV, which is similar to the reported Eg (3.22 eV) value of TiO_2_ and that of MWCNT/TiO_2_ is 2.5 eV, which is slightly red-shifted. Modification of CNT with TiO_2_ mesoporous not only increases the visible-light absorption but also provides a shift in absorption towards longer wavelengths.

The J–V characterization of the DSSCs based on nanostructured MWCNT/TiO_2_ porous electrodes (under illumination of 100 mW cm^−2^) is shown in Fig. 3(a). The short-circuit photocurrent density (Jsc), open-circuit voltage (Voc), fill factor (FF) and the corresponding energy conversion efficiency values (η) are summarized in the inset table of Fig. 3. Compared with a DSSC based on conventional TiO_2_ electrodes with Pt counter electrode, the MWCNT/TiO_2_ layer containing 1 wt.% of CNTs provided an increase of device efficiency, which can be attributed to the enhanced Jsc. The DSSC with the hybrid photoanode and conventional Pt CE exhibited a short-circuit photocurrent (Jsc) of 15.96 mA cm^−2^, open-circuit voltage (Voc) of 0.77 V, FF of 57% and solar conversion efficiency (η) of 7.00%. For the DSSC with pure TiO_2_ mesoporous photoanode and Pt CE fabricated using the same method, the values of Jsc, Voc, FF and η were, 15.68 mA cm^−2^, 0.77 V, 54% and 6.51%, respectively. The hybrid working electrode exhibits an enhanced photocurrent extraction compared with the pure TiO_2_ electrode. The improvement on Jsc can be associated with an enhanced interconnectivity between CNTs and TiO_2_ mesoporous structures. The CNT based photoanode introduces an alternative electrical conduction pathway that facilitates rapid electron transport in the photoelectrode. Nogueira *et al*. showed that the TiO_2_-MWCNT photoanodes were prepared by a direct mixing method. They investigated that the performance of DSSC based on TiO_2_-MWCNT photoanodes is dependent on the amount of MWCNT-COOH addition. When the amount of COOH content increased from 0 to 0.003 wt.%, solar conversion efficiency increased from 2.36% to 3.05^29^. Lee *et al*. also introduced a similar behavior for the working electrode preparation with a small amount of 0.1 wt.% MWCNT^30^. Ho *et al*. indicated that the DSSC with the TiO_2_ photoanode containing 0.1 wt.% of MWCNT resulted in a higher current-density (Jsc = 9.08 mA cm^−2^) and a higher solar conversion efficiency (η = 5.02%)^31^. Park *et al*. used the electro-spun carbon nanotubes/titanium dioxide (CNT/TiO_2_) nanofibers fabricated using a mixture of titanium isopropoxide, MWCNT as photoanode material for DSSCs. DSSCs with CNT/TiO_2_ nanofiber-based working electrodes with the addition of CNTs up to 5 wt.% increased the cell efficiency from 1.43% to 3.39%, while the further addition of CNTs resulted in a decrease of the cell efficiency^32^. Wang *et al*.^33^ showed that the efficiency of DSSCs could be explained using LHE (light harvesting efficiency), electron injection efficiency, and electron collection efficiency, which relate to photo-current density. It was shown that the photo-generated electrons could recombine at the pure TiO_2_/electrolyte interface due to a possible back diffusion of the carriers as illustrated in Fig. 3(d)^34^. However, the use of CNTs in photoanode can quickly transport the photo-generated electrons and reduce the charge recombination probability. By adding an appropriate amount of CNTs to the working electrode, the electron transport characteristics are enhanced, and the collection time of electrons is decreased. As a result, faster collection of electrons results in a decrease in the rate of recombination leading the substantial enhancement in Jsc and solar conversion efficiency. To establish the benefit of the CNT/TiO_2_ working electrodes, we investigated the incident photon-to-current conversion efficiency (IPCE) and electron transport properties of CNT/TiO_2_ hybrid films. The observed improvement of IPCE can be explained by the increased light capture efficiency, electron injection and collection efficiency of the film. Figure 3(b) shows the IPCE spectra of the DSSCs with CNT/TiO_2_ and pure TiO_2_ photoelectrodes as a function of the wavelength. The IPCE at around 520 nm overlaps with the maximum absorption wavelength of the N719 dye. The device with a MWCNT/TiO_2_ based electrode exhibits an enhancement of IPCE in the wavelength range of 350–600 nm, compared with that of the one with pure TiO_2_ electrode. The enhancement of IPCE mainly resulted from the increased electron injection efficiency and LHE of the film. It is also attributed to the enhancement of dye loading via higher surface area of the hybrid film. Devices structure, photo-electrochemical reaction loop, and band alignment MWCNT/TiO_2_ photoanode based DSSC with Pt CE are illustrated in Fig. 3(c,d). TiO_2_ mesoporous structures accept electrons from photo-excited dye N719, and these electrons are transferred to the conduction band of CNTs through the transportation of electrons between TiO_2_ mesoporous structures in a random and zigzag pathway. The electrons in the CNTs’ conduction band, transferred from the TiO_2_ conduction band, can quickly move to the FTO layer without any recombination or back reaction taking place. In contrast, pure TiO_2_ based DSSC have a wide-band structure, hence electrons transferred from TiO_2_ can stay at continuous energy levels near the Fermi level, accelerating recombination of electrons to the dye or back reaction to tri-iodide in the electrolyte.

### Iron Pyrite (FeS_2_) Thin Films for Counter Electrode in DSSC

**Figure 4:**
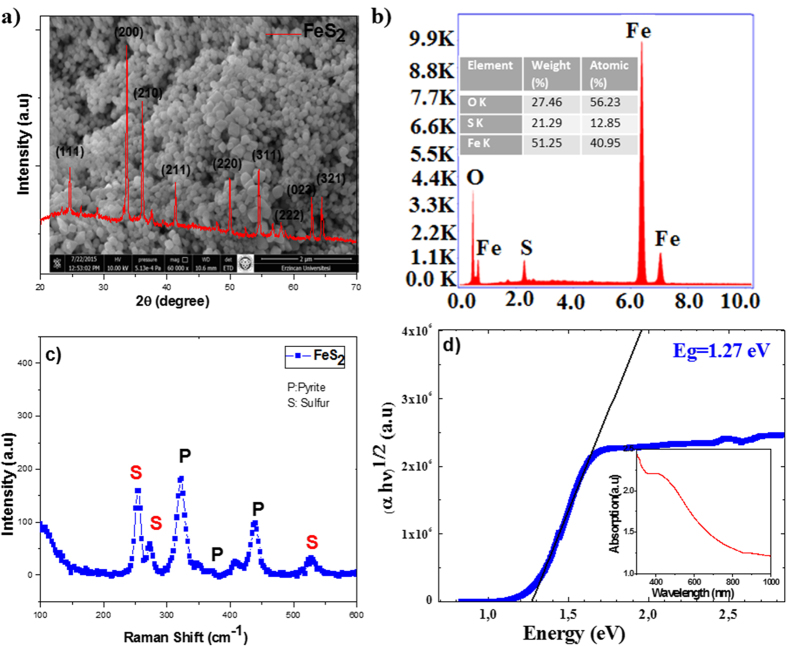
(**a**) XRD pattern of the FeS_2_ particles with corresponding SEM image in inset. (**b)** EDAX analysis (**c)** Raman spectra (**d)** UV-Vis absorbance spectra of FeS_2_.

FeS_2_ thin films were prepared on a FTO coated glass substrate by a simple and cost effective hydrothermal method using (FeCl_3_) 6H_2_O and sulfur as precursor. The concentration of the sulfur precursor was intentionally kept higher to compensate the sulfur loss during the reaction. The thin film was annealed in a sulfur environment at 400 °C for 30 min to crystallize the film with appropriate stoichiometry. Figure 4 shows the SEM, EDAX, XRD, Raman, UV-Vis and surface photo-voltage results of FeS_2_ thin film. The SEM images of FeS_2_ thin film in Fig. 4(a) shows uniform and cubic shaped nanostructures after annealing. The effect of annealing temperature on the thin film can be attributed to the anisotropy of surface free energy and strain energy leading to huge growth during pyrite film formation. As seen in the SEM image, FeS_2_ film contains large homogenously distributed particles between 50–200 nm. The XRD diffraction peaks (Fig. 4a) can be indexed as a pure pyrite cubic phase of FeS_2_ (JCPDS no 42–130), and no obvious impurity peaks were observed. The sharp peaks in the XRD pattern suggest the excellent crystallinity of the as-obtained FeS_2_ thin film. The XRD peaks were recorded as (111), (200), (210), (211), (220), (311), (222), (023) and (321) at 2 ϴ= 24.7, 33.6, 36.1, 41.3, 49.4, 54.3, 57.8, 62.4 and 64.3 degrees, respectively, which is indexed to FeS_2_ cubic structure with high purity. The EDX analysis of the film shows the presence of Iron and sulfur in Fig. 4(b), and experiments showed that the iron concentration increases with temperature. The Fe:S ratio is approximately 33.3:66.6 at room temperature(figure not shown) and 50:30 at 400 °C. This result shows that the deposited FeS_2_ thin film is transformed from FeS_2_ to metallic Fe at 400 °C. The observed sulfur deficiency at elevated temperature is attributed to the low vapor pressure of Sulfur and therefore it sublimates easily at high temperatures. To investigate the chemical bonds and symmetry of molecules, the vibrational information was obtained from Raman spectroscopy. The Raman peaks confirm the phase purity of the film. The active Raman modes of the sample give a symmetric mode (Ag), doubly degenerate (Eg), and three triply degenerate modes. Figure 4(c) shows three Raman peaks at wave-numbers of 330, 370 and 435 cm^−1^ that the characteristic active modes for FeS_2_ are corresponding to the Eg and in phase stretching vibration of Ag, respectively. In the Eg mode, the S atoms are replaced vertically to the dimer axes. The peak at about 520 cm^−1^ corresponds to the coupled vibration and stretching (Tg) modes or their combinations. As a result, the Raman peaks exhibit the FeS_2_ cubic structure and further support the result gathered from XRD analysis. The optical properties of FeS_2_ are investigated by UV–Vis spectroscopy. Figure 4(d) shows the absorption spectra of FeS_2_ thin film. The FeS_2_ film has a strong light absorption in the higher energy region. Figure 4(d) shows the graph of (αhv)^1/2^ vs. photon energy (hv) for FeS_2_ thin film where the “α” is calculated from the absorption spectra utilizing the general formula for indirect allowed transition. The optical band gap energy value of FeS_2_ thin film is obtained as 1.27 eV by fitting the rising portion of the curve in Fig. 4(d). UV-Vis results shows that the size of FeS_2_ particles constituting the film can adjust the local structure and cause a strong change of optical properties. UV-Vis spectra indicated that the FeS_2_ thin film with high density of grain boundaries can create strong photon scattering at interfaces and high optical absorption^35^,^36^.

**Figure 5:**
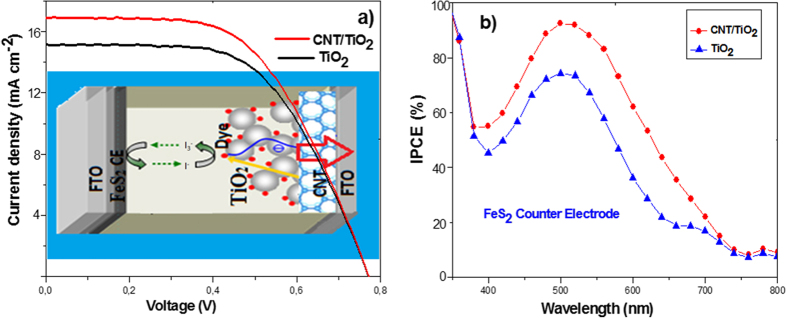
(**a**) Current density-voltage (J-V) characteristics of MWCNT/TiO_2_ and TiO_2_ WEs DSSCs with FeS_2_ thin films as CE. (FeS_2_ film thickness is about 1 μm) Inset: Schematic diagram of a device with MWCNT/TiO_2_ WE. J-V characteristics were obtained under 1-sun AM 1.5G solar irradiance, **(b**) IPCE spectra of the devices.

Figure 5(a) shows the current density-voltage performance of FeS_2_ counter electrode DSSCs under 1-Sun illumination (100 mW/cm^2^, AM 1.5G). The open-circuit voltage (Voc), short-circuit current density (Jsc), and fill factor (FF) of MWCNT/TiO_2_ photoanode based DSSC with FeS_2_ CE are obtained as 0.77 V, 16.86 mA/cm^2^, and 0.56, respectively, thus yielding an energy conversion efficiency of 7.27%. On the other hand, the corresponding values of the pure TiO_2_ photoanode based DSSC with FeS_2_ CE are measured 0.77 V, 15.16 mA/cm^2^, 0.57 and 6.65%, respectively. The improvement of IPCE is mainly the consequence of increased J_SC_, which can be attributed to rapid inter conversion between I_3_^−^ and I^−^, possible reflection of incident photons from FeS_2_ CE layer and appropriate bandgap energy of FeS_2_. Since FeS_2_ has a bandgap energy of 1.27eV, photons can be absorbed in this layer and generated electrons in conduction band may contribute the J_SC_ of DSSC through the transport process either to electrolyte or TiO_2_. It is shown that FeS_2_ CE can also provide a large effective surface area owing to their rough morphology, leading to low charge transfer resistance. IPCE was found to shift upward in DSSCs when FeS_2_ was used as CE in the place of conventional Pt material. IPCE spectra in Fig. 5(b) show typical characteristics of N719 dye at 520 nm. The upward shift of the IPCE curve is an indication of the extra electrons generated in the devices. The shift is consistent with the increment of the Jsc value apparently seen in Fig. 5(a). It has been clearly shown that FeS_2_ materials have a good catalytic activity and have a big potential to be an ideal counter electrode for DSSC applications.

## Conclusion

Multi-walled carbon nanotube (MWCNT) as working electrode on solar conversion efficiency in DSSC is being studied and MWCNT based working electrode showed a distinct improvement in photocurrent compared with the pure TiO_2_ working electrode. The results show that the conversion efficiency of MWCNT/TiO_2_ based cell with conventional Pt CE was improved from 6.51% to 7.00% which is associated with the enhanced interconnectivity between CNT and TiO_2_ mesoporous structures. In addition to the conventional Pt CE we designed a new CE with FeS_2_ nanostructures contained thin film. It was shown that pyrite thin films exhibited higher surface area and good catalytic activity compared to the conventional Pt CE and therefore higher solar conversion efficiency of 7.27% was obtained from MWCNT/TiO_2_ hybrid photoanode based DSSC with FeS_2_ CE. Based on the above results CNT based photoanode grown by a cheap and earth abundant material and catalytically more active FeS_2_ CE grown by a simple and cheap hydrothermal method show a tremendous potential to be utilized in DSSCs industry to enhance the cell efficiency.

## Additional Information

**How to cite this article**: Kilic, B. *et al*. Preparation of Carbon Nanotube/TiO_2_ Mesoporous Hybrid Photoanode with Iron Pyrite (FeS_2_) Thin Films Counter Electrodes for Dye-Sensitized Solar Cell. *Sci. Rep*. **6**, 27052; doi: 10.1038/srep27052 (2016).
